# Using cell-specific late-phase asthma mRNA biomarkers to repurpose drugs that concurrently reverse disease signatures across multiple immune cell-types

**DOI:** 10.1371/journal.pcbi.1014081

**Published:** 2026-04-03

**Authors:** Mingming Zhang, Young Woong Kim, Chen Xi Yang, Gail M. Gauvreau, John Marcus FitzGerald, Louis-Philippe Boulet, Paul M. O’Byrne, Scott J. Tebbutt, Amrit Singh

**Affiliations:** 1 Department of Anesthesiology, Pharmacology and Therapeutics, University of British Columbia, Vancouver, British Columbia, Canada; 2 UBC Centre for Heart Lung Innovation, St. Paul’s Hospital, Vancouver, British Columbia, Canada; 3 Providence Research, Providence Health Care, Vancouver, British Columbia, Canada; 4 Department of Medicine, McMaster University, Hamilton, Ontario, Canada; 5 Division of Respiratory Medicine, Department of Medicine, University of British Columbia, Vancouver, British Columbia, Canada; 6 Centre de Pneumologie de L’Hopital, Université Laval, Québec City, Québec, Canada; 7 PROOF Centre of Excellence, Vancouver, British Columbia, Canada; University of Macau, MACAO

## Abstract

**Background:**

The allergen-induced late-phase asthmatic response (LAR) is used to study the mechanisms and treatment of asthma. We hypothesized that gene-expression (mRNA) biomarkers of the LAR may predict asthma exacerbations and severity.

**Methods:**

Individuals with mild allergic asthma underwent inhaled allergen challenge. We identified a baseline blood-based mRNA biomarker panel predictive of LAR development and combined it with previously reported LAR-associated biomarkers. These LAR-mRNA biomarkers were evaluated in multiple public gene-expression datasets spanning blood, airways, bronchoalveolar lavage fluid (BALF), and induced sputum to assess their ability to discriminate asthma exacerbations and severity. Cellular specificity and allergen responsiveness were examined using public single-cell RNA sequencing datasets from endobronchial biopsies obtained before and after segmental allergen challenge. To identify potential therapeutics, we performed drug-signature matching using a public single-cell perturbation dataset to identify compounds capable of reversing LAR-associated, cell-specific transcriptional changes.

**Results:**

A combined set of 109 LAR-mRNA biomarkers was identified and shown to predict asthma exacerbations, with stronger performance in males than females. Biomarker-based prediction of asthma severity was most robust in BALF and induced sputum compared with blood. Highly predictive transcripts included the RNA-binding proteins *GNAS* and *SF3B1* across multiple sample types. Single-cell analyses revealed that allergen challenge selectively altered the expression of LAR-mRNA biomarkers in T cell populations, including CD8 T cells (*CLIC3*+ and *GZMK*+), CD4 Th17 (*RORA*+) cells, CD4 (*CD40LG*+) T cells, and γδ (*TRDC*+) T cells, with minimal changes in allergic controls. Drug-signature matching identified corticosteroids such as mometasone furoate and prednisolone, as well as the heat shock protein 90 inhibitor AT13387, as compounds predicted to reverse these cell-specific transcriptional signatures.

**Conclusion:**

LAR-mRNA biomarkers reflect an asthma-specific inflammatory state that predisposes individuals to late-phase responses, exacerbations, and progression to more severe disease. These findings link systemic biomarkers to airway cellular mechanisms and nominate therapeutic strategies to modulate allergen-driven inflammation.

## Author summary

Asthma attacks (exacerbations) can be triggered by allergens, but not everyone with allergic asthma responds the same way. In a controlled allergen inhalation challenge, most people develop an early, short-lived narrowing of the airways, while about two-thirds go on to develop a later, longer-lasting response linked to inflammation and increased airway sensitivity. We previously showed that blood gene expression measured before allergen exposure can predict who will develop this late response. In this study, we expanded that work by building a blood-based gene-expression panel that predicts the late-phase response and testing whether these biomarkers also capture patterns seen in real-world asthma exacerbations and more severe disease across multiple public datasets from blood and airway samples. Using single-cell RNA sequencing from airway biopsies, we showed that many of these biomarkers are specifically altered after allergen challenge in people with allergic asthma, especially in T cell subsets, while few changed in allergic controls, supporting asthma-specific regulation. Finally, we matched these cell-specific signatures to drug-perturbation profiles to identify compounds predicted to reverse the allergen-induced patterns, including corticosteroids and an HSP90 inhibitor, generating testable treatment hypotheses.

## Introduction

Allergen inhalation challenge has long been used as a controlled human model to induce airway responses and associated inflammatory processes in individuals with mild allergic asthma [1,2]. Following exposure to a sensitizing allergen, most allergic individuals develop an acute bronchoconstrictive response known as the early-phase asthmatic response (EAR), which typically resolves within 1–3 hours. A substantial subset of individuals subsequently develops a late-phase asthmatic response (LAR), emerging 3–4 hours after allergen exposure and characterized by sustained bronchoconstriction, immune-cell infiltration of the airways, and transient airway hyperresponsiveness [3]. The LAR is of particular clinical relevance, as it reflects inflammatory processes that resemble those observed during asthma exacerbations and more severe disease.

We previously demonstrated that baseline gene-expression profiles measured in whole blood can predict which individuals will go on to develop the LAR following allergen inhalation challenge [4]. These findings suggest that a pre-existing systemic inflammatory state may predispose certain individuals to heightened airway inflammation following allergen exposure. Although allergen challenge represents an experimental model, the molecular programs associated with the LAR may also be operative in real-world settings, where they could contribute to asthma exacerbations and the progression from mild to moderate or severe asthma.

In the present study, we extend this prior work through a comprehensive and integrative computational analysis framework that moves beyond biomarker discovery toward validation, mechanistic interpretation, and therapeutic hypothesis generation. Specifically, we identified a gene-expression biomarker panel from baseline whole-blood samples that predicts development of the LAR after allergen challenge. We integrated this panel with previously reported LAR-associated mRNA biomarkers [4] and systematically evaluated their ability to discriminate asthma exacerbation status and disease severity across multiple publicly available bulk transcriptomic datasets derived from blood, airways, bronchoalveolar lavage fluid (BALF), and induced sputum.

To establish cellular specificity, we leveraged public single-cell RNA-sequencing (scRNAseq) data from endobronchial biopsies obtained from mild allergic asthmatic individuals undergoing segmental allergen challenge [5]. Finally, to connect disease-associated transcriptional programs to potential therapeutics, we repurposed a publicly available scRNAseq drug-perturbation dataset [6] to identify compounds capable of reversing LAR-associated, cell-specific gene-expression signatures across multiple immune cell types simultaneously.

Collectively, this study presents a scalable computational workflow that integrates feature selection, multi-cohort validation, single-cell resolution analyses, and drug-signature matching to generate biologically and clinically relevant hypotheses about asthma pathogenesis and treatment.

## Materials and methods

### Ethics statement

The Institutional Review Boards of the participating institutions, the University of British Columbia (UBC), McMaster University, and Université Laval, approved this study (NCT01113697) and participants provided written informed consent.

### Participant recruitment

A total of 35 participants underwent allergen inhalation challenges and, based on changes in FEV_1_ 3–7 hours post-challenge, were classified as 15 ERs (change in FEV_1_ < 15% fall from baseline) and 20 DRs (change in FEV_1_ ≥ 15% fall from baseline) (Table 1). Participants were required to have mild allergic asthma. Participants were excluded if they smoked, had any other lung or cardiovascular disease, or had a respiratory infection within 6 weeks. All participants had a baseline FEV_1_ ≥ 70% of the predicted value, and a positive methacholine PC_20_ (provocative concentration of methacholine that causes a 20% drop in FEV_1_) test of <16 mg/mL. Participants were not taking maintenance inhaled corticosteroids, and β_2_-agonist use was infrequent and withheld 8 hours prior to spirometry measurements.

**Table 1:** Characteristics of the subjects participating in the allergen inhalation challenge.

Clinical variable		Isolated Early responders (ERs) n = 15	Dual responders (DRs) n = 20	p-value
Percent Female		67%	75%	
BMI (kg/m^2^)		25.4 ± 4.4	25.7 ± 5.3	0.86
Age (years)		28.3 ± 8.0	33.0 ± 13.2	0.23
Baseline FEV_1_ (L)		3.3 ± 0.8	3.1 ± 0.8	0.49
% drop in FEV_1_ during the early-phase (EAR)		−34.5 ± 10.5	−36.9 ± 8.8	0.47
% drop in FEV_1_ during the late-phase (LAR)		−6.6 ± 4.1	−30.4 ± 10.6	**1.9x10** ^**−9**^
Allergen-induced shift in Methacholine PC_20_^a^		1.7 ± 1.7	3.4 ± 1.9	**0.001**
Blood leukocyte (10^9^ cells/L) counts		6.1 ± 1.6	5.6 ± 1.3	0.39
% neutrophils		58.1 ± 9.7	51.8 ± 9.2	0.07
% lymphocytes		29.3 ± 7.3	33.3 ± 9.8	0.21
% monocytes		7.3 ± 1.4	7.8 ± 1.8	0.42
% eosinophils		4.9 ± 3.3	6.4 ± 7.0	0.45
% basophils		0.56 ± 0.34	0.85 ± 0.98	0.29
Allergen (# of individuals)	Cat	10	11	
	Fungus	0	1	
	Grass	2	1	
	HDM	3	5	
	Horse	0	1	
	Ragweed	0	1	
Site (# of individuals)	Laval	10	10	
	McMaster	4	8	
	UBC	1	2	

^a^[Methacholine PC_20_]_pre_/[Methacholine PC_20_]_post_.

Note: summary statistics are expressed as Mean ± SD.

Grass: Grass mix, Orchard Grass, Timothy grass, HDM: House dust mite, *Dermatophagoides (D) Farinae*, *D. Pteronyssinus*.

### Methacholine and allergen challenge

Methacholine PC_20_ was determined one-day prior (pre-) and one-day after (post-) allergen inhalation challenge. The [PC_20_]_pre_/[PC_20_]_post_ was used to determine the allergen induced shift in airway hyperresponsiveness. Skin prick and methacholine challenge testing were used to determine the allergen extract of choice, and the dose of allergen used for the inhalation challenge [7]. Doubling doses of the target allergen were inhaled during 2 minutes of tidal breathing and inhalations were stopped when a fall in FEV_1_ of at least 20% was achieved. Then FEV_1_ was measured at regular intervals up to 7h post-challenge. All participants demonstrated an FEV_1_ drop of at least 20% between 0–2 hours (early-phase asthmatic response, EAR) after allergen inhalation challenge. Participants who demonstrated a maximum drop in FEV_1_ ≥ 15% between 3–7 hours after allergen challenge (late-phase asthmatic response, LAR) were classified as dual responders (DRs). Since the LAR is associated with a 2-fold decrease in methacholine PC20, participants with a greater than 10% fall in FEV1 at 7h post-challenge and an allergen-induced shift in methacholine PC20 greater than or equal to 2 were considered to have experienced LAR after the last spirometric measurement at 7h, and were classified as DRs.

### Blood collection and gene-expression profiling

Blood samples were collected immediately prior to (pre-) allergen inhalation challenge using standard operating protocols at each participating institution (see **S1 Appendix** for details). We used the NanoString nCounter PanCancer Immune Profiling Panel to profile 770 genes related to immune responses to cancer using baseline blood samples. These genes fall into many categories important in the allergic response, such as adaptive, innate and humoral immune response, inflammation, cell-specific genes (B-cells, T-cells), etc. Specifically, these genes consist of 109 cell surface marker genes for 24 cell types, 30 genes for cancer antigens, over 500 genes measuring the immune response and 40 housekeeping genes.

### Data analysis

#### LAR-mRNA biomarkers.

The biomarker panel of the late-phase asthmatic response (LAR-mRNA biomarkers) was identified using sparse Partial Least Squares Discriminant Analysis (sPLSDA) [8] using the mixOmics R library (v6.34.0). In order to identify a LAR-mRNA biomarker panel with a subset of gene-transcripts, we tested a range of sPLSDA models with two components and retaining 5, 10, 15, 20, 25, and 30 gene-transcripts per component using 5-fold cross-validation repeated 20 times (20x5-fold CV). The panel with the fewest number of gene-transcripts and area under the receiver operating characteristics curve (AUC) of greater than 70% was retained. These gene-transcripts were combined with the mRNA biomarkers of the LAR from our previously published study [4]. Enrichment analysis of the list of LAR-mRNA biomarkers was performed using Enrichr [9] (enrichR, R library v3.2), a pathway database (WikiPathways 2024 Human), a cell-type marker database (CellMarker 2024) and a transcription factor database (Rummagene transcription factors). A Benjamini-Hochberg false discovery rate (BH-FDR) of 1% was used for all enrichment analyses.

#### Public bulk gene-expression asthma datasets.

GSE19301: consisted of time-series blood gene-expression profiles from asthmatic individuals [10] (7 with mild persistent, 52 with moderate persistent and 59 with severe persistent asthma). Samples were collected during an initial “quiet” period, an “exacerbation” period and a “follow-up” period. Donors with mild persistent asthma were not included in downstream analyses given the limited sample size. From the 52 donors with moderate (and severe) persistent asthma, 180 (193) samples were collected during a quiet period, 68 (91) during an exacerbation period and 60 (59) during a follow-up period, respectively. Gene-expression data was derived from isolated peripheral blood mononuclear cells and profiled using Affymetrix Human Genome U133A microarrays. The downloaded gene-expression data was previously processed using the Affymetrix MAS 5.0 algorithm (according to the provided metadata). The data was log_2_-transformed and values were below 0 or NAs were replaced with zeros. Probesets mapping to the same gene symbol were averaged across samples.

GSE147878: was a cross-sectional study examining clinical and transcriptomic data from 73 endobronchial biopsies from the European Unbiased Biomarkers for the Prediction of Respiratory Outcomes (U‐BIOPRED) study, and the Australian Priority Research Centre for Healthy Lungs – NOVocastrian Asthma cohort (NOVA) [11–13]. All the subjects were current non-smokers. All the samples underwent transcriptomic profiling using Illumina HT-12 version 4 Beadchips. 13 out of 73 endobronchial biopsies were assigned as healthy controls, 18 were classified as moderate asthmatics and the remaining 42 samples as severe asthmatics. As described in the phenotypic data of the GEO dataset, gene-expression data was previously processed using the GenomeStudio software (Illumina) and the lumi and limma R packages. Gene-expression was already log2-transformed and quantile normalized. No further data transformation was applied. Probesets mapping to the same gene symbol were averaged across samples.

GSE147880: was a cross-sectional study examining clinical and transcriptomic data from 44 induced sputum samples from the European Unbiased Biomarkers for the Prediction of Respiratory Outcomes (U‐BIOPRED) study, and the Australian Priority Research Centre for Healthy Lungs – NOVocastrian Asthma cohort (NOVA) [11–13]. All the subjects were current non-smokers. All the samples underwent transcriptomic profiling using Affymetrix GeneChip Human Genome U133 Plus 2.0 microarray. The 44 sputum samples were classified as 10 healthy controls, 18 mild/moderate and 16 severe asthmatics. We relabelled mild/moderate to moderate for the purpose of this analysis. As described in the phenotypic data of the GEO dataset, gene-expression data was previously processed using the GenomeStudio software (Illumina) and the lumi and limma R packages. Gene-expression was already log2-transformed and quantile normalized. No further data transformation was applied. Probesets mapping to the same gene symbol were averaged across samples.

GSE74986: contains transcripts from bronchoalveolar lavage (BALF) samples collected from moderate asthmatic patients and healthy subjects as controls in the Study of the Mechanisms of Asthma (MAST; clinicaltrials.gov: NCT00595153) [14] study and severe asthmatic patients in the Bronchoscopic Exploratory Research Study of Biomarkers in Corticosteroid-refractory Asthma (BOBCAT) study [15]. BALF was centrifuged to generate cell pellets from 86 subjects. Total RNA was extracted from the cells with the Qiagen RNeasy Kit. The quantity and quality of the total RNA samples were determined with the Bioanalyzer 2100 (Agilent Technologies). Gene-expression was profiled using Agilent Whole Human Genome 4x44K [16]. Among 86 BALF samples, 12 were healthy controls, 28 were moderate asthmatics and 46 were severe asthmatics. The downloaded gene-expression data was previously assessed using the arrayQualityMetrics R package, normalized using the vsn R package and filtered for gene-transcripts with low variation using the genefilter R package (according to the provided metadata). Since data was symmetrically distributed around zero and no further data transformation was applied. Probesets mapping to the same gene symbol were averaged across samples.

GSE69683: consisted of blood gene-expression profiles from 87 healthy controls, 77 patients with moderate asthma, and 334 patients with severe asthma. Transcriptional profiling of whole blood samples was performed using Affymetrix HT HG-U133 + PM Arrays [12]. The downloaded gene-expression data was previously imported into ArrayStudio V8.0 and processed using the default RMA pipeline (according to the provided metadata). The data was log_2_-transformed and values were below 0 or NAs were replaced with zeros. Probesets mapping to the same gene symbol were averaged across samples.

GSE76262: consisted of gene-expression profiles from 21 healthy controls, 25 patients with moderate asthma, and 93 patients with severe asthma from induced sputum. Transcriptional profiling of induced sputum samples was performed using Affymetrix U133 Plus 2.0 PM arrays [17]. As described in the phenotypic data of the GEO dataset, gene-expression data was previously processed using the Affymetrix analysis quality control workflow from arrayanalysis.org and normalized using the Robust Multichip Average. No further data transformation was applied.

GSE161245: transcriptional profiling data of bronchial epithelial cells was obtained via bronchoscopy from a total of 55 subjects among which 16 were healthy controls, 5 were moderate and 17 severe asthma subjects [18]. The RNA samples underwent sequencing using the Illumina NextSeq 500 platform. The downloaded gene-expression data was in raw counts annotated using Ensembl ids. Counts for gene-transcripts mapping to the same gene symbol were summed. The limma-voom library size normalization was performed for each sample [19].

Datasets of the airways (GSE147878 and GSE161245) and induced sputum (GSE76262 and GSE147880) were merged and batch-corrected using the ComBat algorithm (sva R package, v3.52.0). For datasets GSE19301, GSE74986, and GSE161245, where biological sex was not reported, sex was imputed from Y-chromosome gene-expression. Briefly, principal component analysis (PCA) was performed on Y-chromosome gene-expression, followed by k-means clustering (k = 2) on the first two principal components. The cluster exhibiting higher Y-chromosome transcript expression was assigned as male.

### Prediction of asthma exacerbations and severity using LAR-mRNA biomarkers

**Fig 1:**
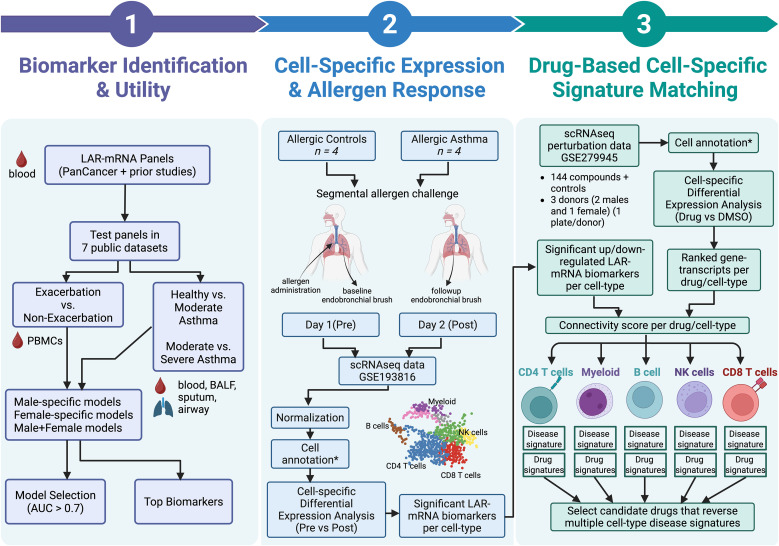
Study workflow. The workflow comprised three stages. In Stage 1, LAR-mRNA biomarkers were selected and used to develop random forest and PLS-DA classification models in publicly available gene-expression datasets of asthma exacerbations and disease severity derived from airways, bronchoalveolar lavage fluid, blood, and induced sputum. Classification performance across clinical comparisons (exacerbation vs quiet or follow-up, healthy control vs moderate or severe asthma, and moderate vs severe asthma) was evaluated using 5-fold cross-validation repeated 20 times, with all analyses additionally stratified by sex. In Stage 2, the cellular specificity of the LAR-mRNA biomarkers was assessed using a public single-cell RNA-sequencing dataset from endobronchial biopsies collected before and after segmental allergen challenge, with cell-type–specific differential expression analyses used to determine changes in average cellular expression following allergen exposure. In Stage 3, a public single-cell RNA-sequencing drug-perturbation dataset from peripheral blood mononuclear cells was used to derive cell-type–specific drug signatures relative to dimethylsulfoxide, and significant up- and down-regulated LAR-mRNA biomarkers were compared with corresponding drug signatures using a connectivity score to identify compounds predicted to reverse allergen-induced transcriptional changes. *cell annotation: cell labels were provided in the downloaded datasets. Created in BioRender.com.

We evaluated the resulting set of LAR-mRNA biomarkers in seven public gene-expression datasets to discriminate between exacerbation and non-exacerbation samples, healthy controls and moderate asthma samples, and moderate and severe asthma samples in airway (endobronchial biopsy), bronchoalveolar lavage (BALF), induced sputum and blood samples (**Fig 1A**). We developed both random forest (ranger R package v0.17.0) and PLSDA (mixOmics R package v6.34.0) models based on all LAR-mRNA biomarkers across different groups and sample-types, and classification performance was computed using 20x-5-fold CV. We also developed sex-specific models to assess whether the classification performance was different between males and females. For each model, we computed feature importance scores (node purity for random forest models and variable importance in projection for PLS-DA models) and converted these values to percentage ranks, such that the feature with the highest importance score was assigned a rank of 1 and the feature with the lowest importance score was assigned a rank of 0. The average rank per biomarker was computed across models with AUC > 0.70. For top ranked biomarkers, we restricted our interpretation of biomarkers with known biological processes based on gene set analysis using Enrichr [20] using the 2025 gene ontology biological process.

### Cell-specific expression of LAR-mRNA biomarkers and response to allergen challenge

We performed cell-specific differential gene-expression using a single-cell gene-expression RNA-sequencing (scRNAseq) dataset (GSE193816) from endobronchial biopsies from individuals with allergic asthma (n = 4) and allergic controls (n = 4) undergoing segmental allergen challenge [5] (**Fig 1B**). Individuals with mild allergic asthma were allergic to either cat allergen or house dust mite (positive skin prick test). Their forced vital capacity (FVC) was ≥ 75% percent predicted and their methacholine challenge test was positive. Allergic controls had no history of asthma, and were allergic to either cat allergen or house dust mite (positive skin prick test). Their FVC was ≥ 75% percent predicted and their methacholine challenge test was negative. Endobronchial samples (n = 4 from each group) were obtained pre- and post-allergen challenge.

The downloaded scRNAseq data for all cells, mononuclear phagocytes (MNP) and T cells were in AnnData format. Cell annotations for the major cell-types and MNP and T cell subtypes were available with the downloaded data. The SingleCellExperiment R-library (v1.26.0) was used to convert the data to a single cell experiment class. The data was normalized by cell-specific library sizes, and then transformed using log_2_(*x* + 1) where *x* is the gene-transcript count. The logcounts were averaged per cell, per sample, prior to cell-specific differential expression analysis (comparing pre- and post-allergen challenge samples) using limma-trend from the muscat R library (v1.18.0). Results were restricted to LAR-mRNA biomarkers and significance was based on a BH-FDR threshold of 20%.

### Reversing cell-specific expression of LAR-mRNA biomarkers using drug-based signature-matching

We also downloaded a single-cell perturbation dataset [21] (GSE279945), in which peripheral blood mononuclear cells (PBMCs) from three donors (2 male, 1 female) were seeded onto three 96-well plates. The PBMCs were exposed to 144 small molecule compounds, 2 positive controls (dabrafenib, belinostat) and a negative control (dimethylsulfoxide, DMSO). Cell annotations for major cell-types, such as CD4 T cells, CD8 T cells, Myeloid cells, B cells and Natural Killer (NK) cells, were available with the downloaded data. The count data was normalized by cell-specific library size, then transformed using log_2_(*x* + 1) where *x* is the gene-transcript count. The logcounts were averaged per cell, per sample, prior to cell-specific differential expression analysis using limma-trend (limma 3.60.6). We applied a regression model consisting of all 144 compounds as predictors (using DMSO as the reference) to each gene-transcript per cell-type. The product of the log_2_ fold-change and -log_10_(*P-value*) was used to rank gene-transcripts for a given drug and cell-type.

An enrichment score was determined by comparing the significant up-/down-regulated LAR-mRNA biomarkers (from the scRNAseq asthma exacerbation dataset) with a ranked list of gene-transcripts for each drug and cell-type. For each cell-type, the difference between the up and down enrichment score was computed, and termed the connectivity score (**Fig 1C**).

## Results

### PanCancer biomarker panel of the late-phase asthmatic response

**Fig 2:**
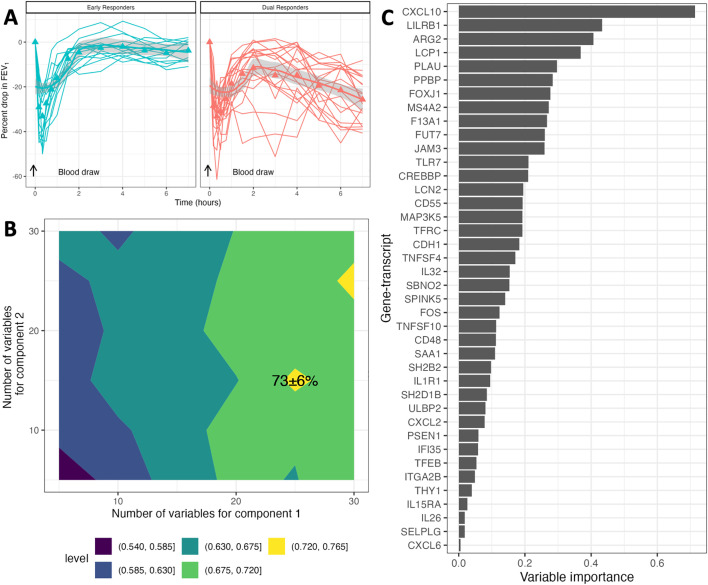
PanCancer gene-expression biomarker panel of the late-phase response. **A.** Blood was collected prior to the allergen inhalation challenge. The allergen inhalation challenge was performed at 0 hours. FEV_1_ was measured using spirometry from 0-7 hours at regular intervals. A 15% drop in FEV_1_ during the late-phase asthmatic response (LAR) was mainly used to characterize participants as dual responders. **B.** Parameter tuning of the sPLSDA classification model to select the number of variables for each component that maximized the 20x-5-fold cross-validation AUC. **C.** Variable importance based on the absolute value of the sPLSDA model coefficients for each gene biomarker.

Following allergen inhalation, all participants developed an EAR whereas participants with a LAR (dual responders DRs) developed a subsequent decline in lung function between 3–7 hours post inhalation challenge. The fall in FEV_1_ during was the late-phase was 30.4 ± 10.6% for DRs compared to -6.6 ± 4.1% for ERs (p < 0.01, **Fig 2A**). Two DRs had a maximum drop in FEV_1_ of -12.6% and -14.9% and thus did not meet the -15% drop criteria; however, since the allergen-induced shift in methacholine PC_20_ was more than 2 doubling doses, they were classified as DRs (**Fig A in** **S1 Appendix**). The allergen-induced shift in methacholine PC_20_ was over 3 times greater in DRs compared to ERs (**Table 1**). No demographic variables or complete blood counts and differentials were statistically different between ERs and DRs; however, the percentage of neutrophils was marginally, but not significantly, greater in ERs compared to DRs (**Table 1**).

After filtering out low abundant transcripts and housekeeping genes, 600 out of the 770 genes were retained. With hyperparameter tuning of the sPLSDA model we obtained a biomarker panel of 40 gene-transcripts (25 and 15 gene-transcripts for components 1 and 2, respectively with no overlaps), which had an average AUC of 73 ± 6% using a 20x5-fold CV (**Fig 2B**). **Fig 2C** depicts the coefficient weights (absolute value) of each gene-biomarker in the sPLSDA model, with C-X-C motif chemokine ligand (*CXCL10)*, leukocyte immunoglobulin like receptor B1 (*LILRB1*) and arginase 2 (*ARG2*) identified as the top three gene-transcripts.

### Biological assessment of the mRNA biomarkers of the late-phase asthmatic response

**Fig 3:**
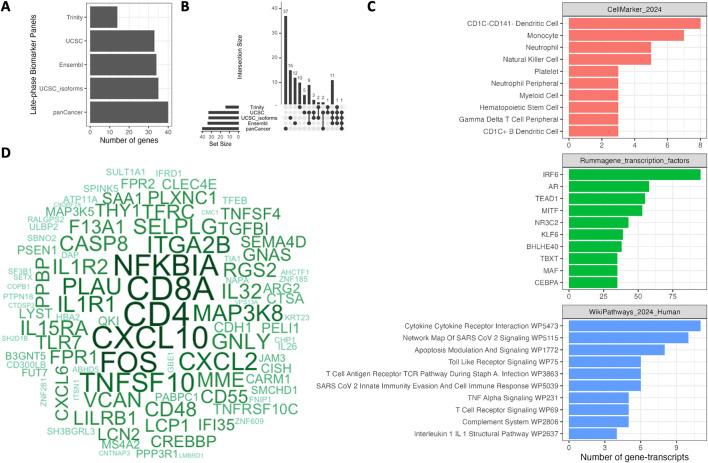
Enrichment analysis of all gene-expression biomarkers of the late-phase response of the current and published studies. **A.** Number of gene-transcripts across the five late-phase biomarkers panels. **B.** Overlap in gene-transcript biomarkers across the five late-phase biomarker panels. **C.** Top 10 gene sets for three EnrichR databases (cell-type markers, transcription factors and pathways) given an adjusted p-value cut-off of 1% and the number of gene-transcript biomarkers in each geneset. **D.** Frequently occurring gene-transcript biomarkers in the significant gene sets identified in **C.**

The PanCancer LAR-mRNA biomarker panel consisting of 40 gene-transcripts was combined with four other published LAR-mRNA biomarker panels (33, 35, 34 and 14 gene-transcripts in the UCSC genes, UCSC gene-isoforms, Ensembl and Trinity panels). Across five LAR-mRNA biomarker panels, 156 biomarkers were identified in total (**Fig 3A**). After removing the duplicated biomarkers, 109 biomarkers were obtained for the combined LAR-mRNA biomarker panel. All of these panels were compared to illustrate the overlapping biomarkers (**Fig 3B**). 37 out of 40 genes from the PanCancer panel were independent biomarkers whereas 3 overlapped with the other panels. 15 independent genes were found in the UCSC-isoforms panel, 12 in the Ensembl panel and 10 in the Trinity panel. Nine genes were seen overlapping in both UCSC-isoforms and Ensembl panels. 11 genes were detected overlapping in three panels (UCSC-isoforms, Ensembl and UCSC). Three EnrichR databases were used to explore how the biomarkers found in the five panels related to possible cell types (CellMarker_2024 filtered for blood cells only), transcription factors (Rummagene_transcription_factors) and pathways (Wikipathways_2024_Human) using gene set analysis. Gene set analysis identified 32 significant cell-types, 190 transcription factors and 10 pathways at an FDR cut-off of 1%. **Fig 3C** depicts the top 10 cell-types, transcription factors and pathways. The late-phase biomarkers were most related to CD1C-CD141 dendritic cells followed by monocytes and neutrophils. IRF6 was the top transcription factor. The top biological pathway was the cytokine-cytokine receptor interaction pathway. The SARS-CoV-2 signaling pathway and apoptosis modulation and signaling pathways were also significantly enriched. Amongst the significant gene sets, *CD4, CD8, CXCL10, NFKBIA,* and *FOS* were the most frequently occurring genes (**Fig 3D**).

### Blood-based LAR-mRNA biomarkers are associated with asthma exacerbations and severity

**Fig 4:**
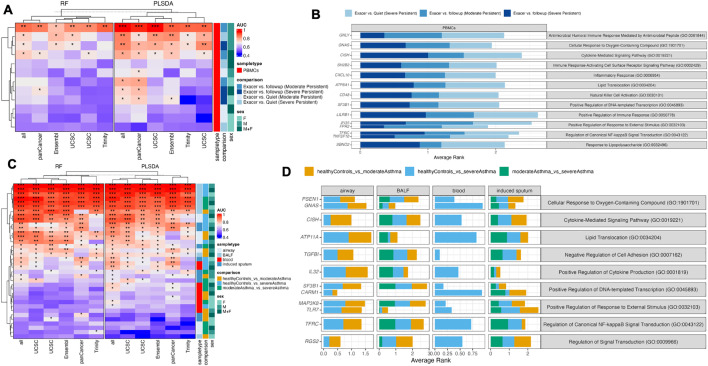
Application of LAR-mRNA biomarker panels to public real-world asthma datasets. **A.** Heatmap of AUCs for RF and PLSDA models fixed to LAR-mRNA biomarkers predicting asthma exacerbations [compared to baseline (quiet) or follow-up]. **B.** Gene-transcripts with the largest importance scores in the exacerbation models (with AUC > 0.70) with annotated gene ontology biological processes. **C.** Heatmap of AUCs for RF and PLSDA models fixed to LAR-mRNA biomarkers predicting asthma severity. **D.** Gene-transcripts with the largest importance scores in the severity models (with AUC > 0.70) with annotated gene ontology biological processes. * indicates AUC > 0.7, **indicates AUC > 0.8 and ***indicates AUC > 0.9.

Using public time-series gene-expression data from peripheral blood mononuclear cells of asthmatic individuals, classification models restricted to different LAR-mRNA biomarker panels (and combined) were used to discriminate samples collected during an exacerbation time-period from samples collected at baseline (called quiet) or follow-up time-period. Sex was imputed using gene-expression from the Y-chromosome (see methods for details). The accuracy of this approach was approximately 72%, tested using the gene-expression data with available sex data (**Fig B in** **S1 Appendix**). **Fig 4A** depicts the AUC (estimated using 20x5-fold CV) of different LAR-mRNA biomarker panels assessed across various comparisons, sex and methods [6 panels x 4 comparisons x 3 (male/female/combined) x 2 methods = 144 models]. 15 models predicted exacerbations with an AUC > 0.70 using both RF and PLSDA. Interestingly 11 of 15 models were specific to males only, suggesting sex-specific differences with respect to asthma exacerbations. Across all models with an AUC > 0.70, gene-transcripts with the highest importance were associated with biological processes such as positive regulation of response to external stimulus (*IFI35* and *FPR2),* and regulation of canonical NF-kappaB signal transduction *(TFRC* and *TNFSF10)* (**Fig 4B**). To further elucidate the predictive performance of the male-specific biomarker panels, we examined the two highest-ranking transcripts from male-specific models (AUC > 0.70) across multiple comparisons (exacerbation vs. quiet, exacerbation vs. follow-up) and asthma severities (moderate and severe persistent). Interestingly, PBMC expression of N-ethylmaleimide-sensitive factor attachment protein (NAPA), cathepsin A (CTSA), cytokine-inducible SH2-containing protein (CISH), and leukocyte immunoglobulin-like receptor B1 (LILRB1) increased during the exacerbation period and decreased during follow-up in male subjects only (**Fig C in** **S1 Appendix**).

We also evaluated the utility of the late-phase mRNA biomarkers to predict asthma severity. We collected public gene-expression datasets from airways (bronchial epithelial cells), bronchoalveolar lavage fluid (BALF), induced sputum and blood. Restricting each dataset to the LAR-mRNA biomarkers (and combined), we determined the performance of classification models to discriminate moderate and severe asthma from healthy controls and between moderate and severe asthma using 20x5-fold CV. Further we stratified this analysis based on sample type and sex (**Fig 4C**). In total, 432 models were generated (4 sample types × 3 comparisons × 6 biomarker panels × male, female, and combined cohorts × 2 classifiers), of which 103 achieved an AUC greater than 0.70 using either RF or PLS-DA. 77 of the 103 models consisted of models comparing healthy controls and moderate or severe asthma. The remaining 26 models which could differentiate moderate from severe asthma were specific to BALF and induced sputum sample types. Models based on PanCancer biomarkers performed competitively with previously identified LAR-mRNA biomarkers. Gene-transcripts with high importance scores belonged to similar pathways as the asthma exacerbation models but also included additional molecular functions such as negative regulation of cell adhesion and positive regulation of cytokine production (**Fig 4D**).

### Cell-specific expression of LAR-mRNA biomarkers in response to allergen challenge

**Fig 5:**
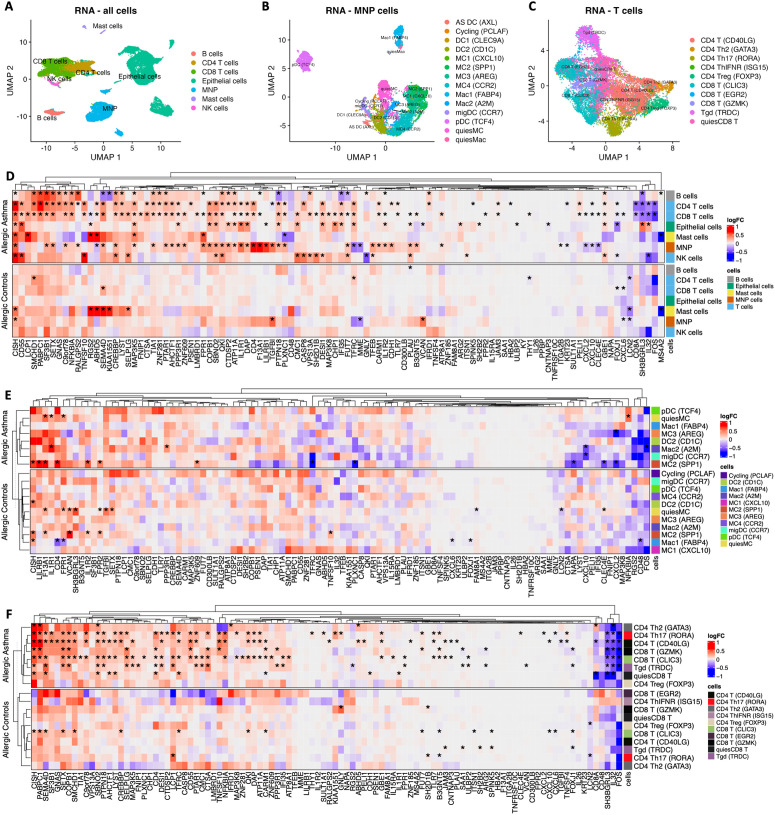
Cell-specific differential expression analysis of LAR-mRNA biomarkers. **A.** Dimension reduction plot of the major cell-types identified in the endobronchial biopsies. **B.** Dimension reduction plot of the mononuclear phagocyte (MNP) subtypes identified in the endobronchial biopsies. **C.** Dimension reduction plot of the T-cell subtypes identified in the endobronchial biopsies. **D.** Log_2_-fold-change for all LAR-mRNA biomarkers across the major cell-types in individuals with allergic asthma and allergic controls (average expression of a given gene-transcript in a given cell-type after allergen challenge minus the average expression at baseline). **E.** Log_2_-fold-change for all LAR-mRNA biomarkers across the MNP subtypes in individuals with allergic asthma and allergic controls (average expression of a given gene-transcript in a given cell-type after allergen challenge minus the average expression at baseline). **F.** Log_2_-fold-change for all LAR-mRNA biomarkers across the T-cell subtypes in individuals with allergic asthma and allergic controls (average expression of a given gene-transcript in a given cell-type after allergen challenge minus the average expression at baseline). *indicates gene-transcripts with a significant changes in average cell-expression due to allergen challenge (BH-FDR < 20%).

While the cell-type enrichment analysis revealed the cell-types where the LAR-mRNA biomarkers are expressed, we sought to determine whether cell-specific expression was modified after allergen challenge. Using scRNAseq data from cell-types in endobronchial biopsies before and after segmental challenge (**Fig D in** **S1 Appendix**), we identified significant cell-specific gene-transcripts in response to allergen exposure (see **Table A in** **S1 Appendix**). Levels of most LAR-mRNA biomarkers were significantly altered following allergen challenge in individuals with allergic asthma, whereas no comparable post-challenge changes were observed in allergic controls (**Fig 5A**; * indicates significant gene-transcripts per cell-type). Further, most LAR-mRNA biomarkers were up-regulated after allergen challenge, with 55 and 42 up-regulated LAR-mRNA biomarkers in CD8 and CD4 T cells, respectively. Dissecting the T cell and MNP populations further indicated that the LAR-mRNA biomarkers were significantly altered in T cell subtypes but not MNP subtypes (**Fig 5B-5C**). T cell subtypes with the greatest number of differentially expressed LAR-mRNA biomarkers included CD8 T cells (CLIC3+ and GZMK+), CD4 Th17 (RORA+) cells, CD4 (CD40LG+) T cells and γδ T cells (Tgd), with most biomarkers being up-regulated (**Table A in** **S1 Appendix**).

### Signature-matching identifies drugs that may attenuate asthma exacerbations

**Fig 6:**
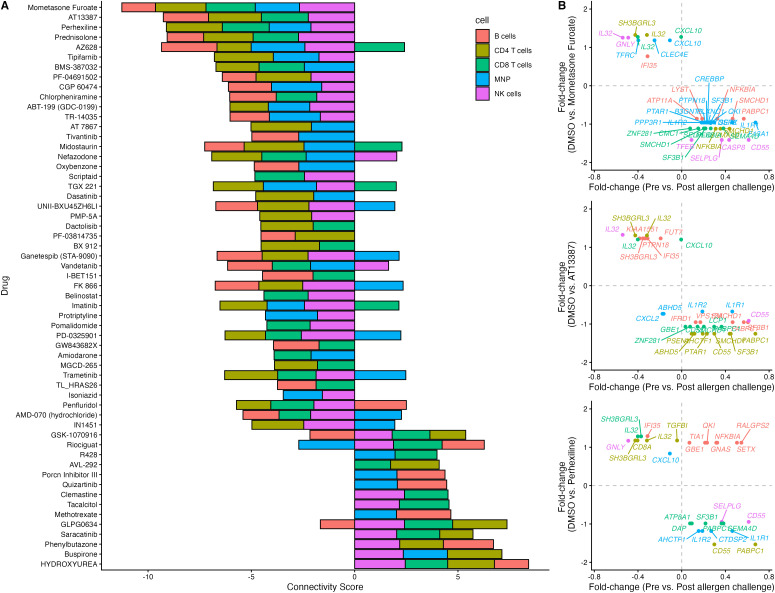
Signature-matching identifies drugs that can reverse the gene-expression changes of the LAR-mRNA biomarkers in response to allergen inhalation. **A.** Connectivity score for each drug and cell-type combination based on an enrichment analysis of significant (BH-FDR < 20%) up- and down-regulated LAR-mRNA biomarkers. A negative score implies the drug alters gene-expression in the opposite direction to the allergen-change in the same cell-type, whereas a positive score indicates the drug alters gene-expression in the same direction. **B.** The fold-change for cell-specific LAR-mRNA biomarkers is shown between the drug (y-axis) and allergen-challenge (x-axis). A negative association was observed for each cell-type for the top 3 drug candidates (mometasone furoate, AT13387 and prednisolone).

In order to identify potential therapeutics that may be used to suppress the cell-specific inflammatory response observed in various immune cell subtypes, we used a public drug perturbation scRNAseq dataset developed by exposing various PBMCs to 144 compounds [21]. We computed a connectivity score for each cell-type drug combination, where a negative score implies that a drug (relative to DMSO) has the opposite effect as allergen exposure (relative to baseline) in the same immune cell-type, whereas a positive score implies that the drug has the same effect. **Fig 6A** depicts the connectivity scores (greater than an absolute value of 3) for each drug and cell-type combination. Mometasone furoate has a negative connectivity score in all cell-types whereas hydroxyurea has a connectivity score greater than zero in all cell-types. **Fig 6B** depicts the fold-changes of the top three drug candidates with a negative score; mometasone furoate, AT13387 and prednisolone. Interestingly, these include corticosteroids which are already known to suppress the inflammatory response observed in allergic asthmatics after allergen challenge.

## Discussion

In this study we identified a gene-expression biomarker panel to predict a subject’s response to allergen challenge and identify participants who experience the late-phase asthmatic response. Combined with existing gene-expression biomarkers of the LAR [4], we were able to show the utility of these biomarkers in discriminating exacerbation from non-exacerbation and severe from moderate asthma. Further we demonstrated that most LAR-mRNA biomarkers are up-regulated in various immune cell subtypes and that existing drugs may be used to suppress this inflammatory response. These results suggest that molecular changes observed during the LAR in mild asthma may play an important role during asthma exacerbations and progression to severe asthma, which may offer an avenue for new asthma treatments.

A central contribution of this work lies not in the development of new algorithms, but in the systematic integration of existing computational methods into a cohesive analysis pipeline. By linking sparse feature selection, multi-cohort validation, single-cell resolution analyses, and drug-perturbation signature matching, we were able to move from biomarker identification to mechanistic insight and therapeutic hypothesis generation. This integrative framework highlights how publicly available transcriptomic and perturbation datasets can be repurposed to address complex, multi-scale questions in asthma biology.

PanCancer biomarkers identified as part of this study and in combination with existing LAR-mRNA biomarkers performed well in discriminating exacerbation from non-exacerbation samples.

Male-specific models performed better at predicting asthma exacerbations than female-specific models regardless of asthma severity. Gene-transcripts with the highest importance scores in the well-performing male-specific panels includes transcripts such as *NAPA, CTSA, CISH* and *LILRB1* which increased in during exacerbations and decreased during follow-up in males only. NAPA is involved in vesicle trafficking and promotes extracellular matrix adhesion [22], processes that may facilitate immune-cell migration in asthma. CTSA encodes a lysosomal carboxypeptidase [23] that regulates the processing of endogenous peptides, including bradykinin, substance P, oxytocin, angiotensin I, and endothelin-1 [24]. CISH functions as a negative regulator of T-cell receptor signaling [25] and cytokine-mediated immune responses, while LILRB1 is an inhibitory leukocyte immunoglobulin-like receptor that suppresses antigen-presenting cell activation and downstream T-cell responses [26]. Together, these findings suggest that male exacerbations are accompanied by the induction of transcriptional feedback and inhibitory immune pathways, which may constrain sustained T-cell and cytokine signaling. In contrast, the absence of similar transcriptional regulation in females may permit more persistent adaptive immune activation, potentially contributing to greater exacerbation severity. Previous studies have also shown sex differences in the frequency of asthma exacerbations and severity of airflow obstruction [27].

With respect to asthma severity, the LAR-mRNA biomarker panels were effective in discriminating healthy controls from severe asthmatics but not from moderate asthmatics. Although the LAR-mRNA biomarkers were initially identified in blood, they performed substantially better in transcriptional signatures derived from BALF and induced sputum, where they also distinguished moderate from severe asthma. In the airways (mostly likely epithelial cells only), the LAR-mRNA could discriminate between healthy controls and moderate and severe asthmatics, but not between moderate and severe asthmatics. This is likely due to the fact that the LAR-mRNA biomarkers were identified using blood samples. The LAR-mRNA biomarkers with the highest importance scores included previously known gene-transcripts associated with asthma severity such as *IL32* [28], *TLR7* [29], *TFRC* [30], *RGS2* [31], *CISH* [32], and *MAP3K8* [33]. On the other hand, gene-transcripts with high importance scores but limited prior association with asthma such as guanine nucleotide binding protein, alpha stimulating (GNAS) and Splicing factor 3B subunit 1 (*SF3B1*) were also identified. The single-cell data suggested that *GNAS* was up-regulated in B cells and CD4 T cells, whereas *SF3B1* was up-regulated in B cells, CD4 T cells and CD8 T cells and MNP cells after allergen challenge. GNAS increases cyclic AMP leading to bronchodilation in airway smooth muscle cells [34] and Protein kinase A activation which inhibits T-cell activation [35]. Up-regulation of *SF3B1* suggests increased alternative splicing after allergen challenge. Mutations in *SF3B1* has been shown to up-regulate pro-inflammatory genes such as *S100A8* and the activate NF-κB pathway [36]. Thus, upregulation of *GNAS* and *SF3B1* after allergen challenge likely reflects late-phase counter-regulatory signaling and increased RNA-processing demand during immune activation and epithelial repair. To our knowledge there is no known association of these transcripts with asthma exacerbations or severity.

Lastly, we identified drug candidates predicted to reverse the cell-type–specific transcriptional signatures of the LAR-mRNA biomarkers induced by allergen challenge. The identification of corticosteroids, including mometasone furoate and prednisolone, was expected given their established role as first-line therapies for asthma. In contrast, the heat shock protein 90 (HSP90) inhibitor AT13387 is not currently used in asthma treatment, nor has it been evaluated in asthma-focused clinical trials. Nevertheless, accumulating evidence supports a role for HSP90 in asthma-relevant pathophysiology. HSP90 is a core molecular chaperone that stabilizes a broad set of intracellular signalling proteins, including kinases and transcriptional regulators that are shared across immune cell types, such as NF-κB and STAT family members, and modulates inflammatory signalling programs [37–39]. Pharmacologic HSP90 inhibition has been shown to modulate airway epithelial inflammatory responses by altering NF-κB pathway activity and blocking cytokine-induced transcriptional programmes in epithelial models [40]. HSP90 inhibition during an ovalbumin challenge in a mouse model prevented leukocyte infiltration and decreased the number of neutrophils [41]. HSP90 inhibition has been shown to induce transcriptional upregulation of HSP70 and HSP27 in PBMCs, both of which are downstream targets of HSF1 [42]. Although HSP90 inhibitors have primarily been developed in oncology, where dose-limiting toxicities have limited their use as monotherapies, they have demonstrated efficacy in combination regimens through synergistic antitumour effects [43]. Notably, the HSP90 inhibitor pimitespib was recently approved in Japan for the treatment of gastrointestinal stromal tumours after demonstrating clinical benefit [44]. Together, these findings support a potential therapeutic role for HSP90 inhibition in asthma.

Despite the breadth of this study, the major limitations are related to the suitability of some of the public datasets used. For example, the LAR-mRNA biomarkers were identified using whole blood, however the asthma exacerbations dataset was restricted to PBMCs which exclude cell-types such as neutrophils, eosinophils and basophils. Some LAR-mRNA biomarkers were neutrophil-specific, which may have resulted in reduced classification performance in being able to predict asthma exacerbations. The drug-perturbation datasets were also limited to PBMCs and were limited by the number of drug compounds that were investigated (mostly anti-cancer drugs).

In summary, we have demonstrated that transcriptional signatures in the blood of asthmatic individuals who elicit the LAR are similar to the transcriptional signatures observed during asthma exacerbations and as asthma becomes more severe. Further, allergen challenge significantly alters these signatures in a cell-specific manner, and can be targeted using therapeutics that reverse these transcriptional profiles.

## Supporting information

S1 Appendix Text. Blood collection and NanoString gene-expression profiling. Methods describing blood collection, RNA purification, RNA quantification and quality assessment, and NanoString nCounter gene-expression profiling and quality-control procedures. **Fig A.** Relationship between the maximum drop in FEV_1_ during the late-phase and the allergen-induced shift. The scatter plot depicts the maximum drop in FEV_1_ during the late-phase (3–7 hours post-allergen challenge) and the corresponding allergen-induced shift (AIS, ratio between the PC20 from one-day prior to allergen-challenge and the PC20 from one-day after allergen-challenge). A subject is classified as a dual responder if the maximum drop in FEV_1_ during the late-phase is greater than 15%. However, if the maximum drop is between 10–15%, but the AIS is greater than 2, the participant is also classified as a dual responder. **Fig B.** Accuracy of sex imputation in GEO datasets with available biological sex information. Principal component analysis was performed on Y-chromosome gene-expression, followed by k-means clustering (k = 2) on the first two principal components. The cluster exhibiting higher Y-chromosome transcript expression was assigned as male. **Fig C.** Top-ranked biomarkers in male-specific models. The two highest-ranking transcripts from male-specific models (AUC > 0.70) were examined across multiple comparisons (exacerbation vs quiet and exacerbation vs follow-up) and asthma severities (moderate and severe persistent). Notably, PBMC expression of N-ethylmaleimide-sensitive factor attachment protein (*NAPA*), cathepsin A (*CTSA*), cytokine-inducible SH2-containing protein (*CISH*), and leukocyte immunoglobulin-like receptor B1 (*LILRB1*) increased during exacerbations and decreased during follow-up exclusively in male subjects. **Fig D.** Proportion of cell-types before and after allergen challenge in allergic controls and asthmatics. AC: Allergic Controls, AA: Allergic Asthma, Bln: baseline, Ag: allergen. **Table A.** Number of significant cell-specific genes at a BH-FDR < 20%.
(DOCX)
