# Supplementary material for: Using cell-specific late-phase asthma mRNA biomarkers to repurpose drugs that concurrently reverse disease signatures across multiple immune cell-types
Source: PLoS Comput Biol. 2026 Apr 3;22(4):e1014081. doi: 10.1371/journal.pcbi.1014081 (PMC13068335; doi:10.1371/journal.pcbi.1014081)
Supplement: S1 Appendix — Blood collection and NanoString gene-expression profiling. Methods describing blood collection, RNA purification, RNA quantification and quality assessment, and NanoString nCounter gene-expression profiling and quality-control procedures. Fig A. Relationship between the maximum drop in FEV1 during the late-phase and the allergen-induced shift. The scatter plot depicts the maximum drop in FEV1 during the late-phase (3–7 hours post-allergen challenge) and the corresponding allergen-induced shift (AIS, ratio between the PC20 from one-day prior to allergen-challenge and the PC20 from one-day after allergen-challenge). A subject is classified as a dual responder if the maximum drop in FEV1 during the late-phase is greater than 15%. However, if the maximum drop is between 10–15%, but the AIS is greater than 2, the participant is also classified as a dual responder. Fig B. Accuracy of sex imputation in GEO datasets with available biological sex information. Principal component analysis was performed on Y-chromosome gene-expression, followed by k-means clustering (k = 2) on the first two principal components. The cluster exhibiting higher Y-chromosome transcript expression was assigned as male. Fig C. Top-ranked biomarkers in male-specific models. The two highest-ranking transcripts from male-specific models (AUC > 0.70) were examined across multiple comparisons (exacerbation vs quiet and exacerbation vs follow-up) and asthma severities (moderate and severe persistent). Notably, PBMC expression of N-ethylmaleimide-sensitive factor attachment protein (NAPA), cathepsin A (CTSA), cytokine-inducible SH2-containing protein (CISH), and leukocyte immunoglobulin-like receptor B1 (LILRB1) increased during exacerbations and decreased during follow-up exclusively in male subjects. Fig D. Proportion of cell-types before and after allergen challenge in allergic controls and asthmatics. AC: Allergic Controls, AA: Allergic Asthma, Bln: baseline, Ag: allergen. Table A. Number of significan [file pcbi.1014081.s001.docx]

**Supporting information**

**Blood collection**

2.5 mL of blood was collected in PAXgene Blood RNA tubes which contain an additive that lyses red blood cells and stabilizes intracellular RNA preventing both degradation and changes in gene expression profiling due to sample handling. These samples were kept at -80°C prior to shipment to the Tebbutt laboratory in Vancouver, Canada. After overnight thawing of PAXgene tubes, total RNA was purified using the PAXgene Blood RNA kit (PreAnalytiX-Qiagen, Germany) using 5 mL of the PAXgene solution (~2.5 mL of blood + 6.4 mL of stabilizing reagent). RNA concentrations were determined using a NanoDrop 8000 Spectrophotometer (Thermo Scientific, Wilmington, DE, USA). RNA quality was assessed using the Agilent 2100 Bioanalyzer following the RNA 6000 Nano kit protocol (Agilent Technologies, Santa Clara, CA, USA).

**Nanostring gene-expression profiling**

We profiled the expression of the 770 panCancer genes using the nCounter® gene expression assay (NanoString Technologies, Seattle). 100 ng of sample RNA (for each of the 36 samples) was used. Briefly, 130 μL of hybridization buffer was added to the reporter codeset (PanCancer Immune Profiling Panel) to create the master mix. 20 μL of the master mix was added to 5 μL of the RNA sample in a strip tube of 12 tubes (3 sets in total for 36 samples from 35 subjects). 5 μL of the capture probeset was added to each tube and then placed at 65°C in a pre-heated thermocycler for 18 hours. Each strip tube was processed with the nCounter Prep-station which purified and immobilized samples onto the nCounter cartridge. The same cartridge was then scanned using the nCounter® Digital Analyzer, which counted the target molecules at 555 fields of view (FOVs). We did quality assessment of the PanCancer NanoString data in RStudio following the data analysis guide by NanoString Technologies (Seattle, USA).

**Supplementary Figures**


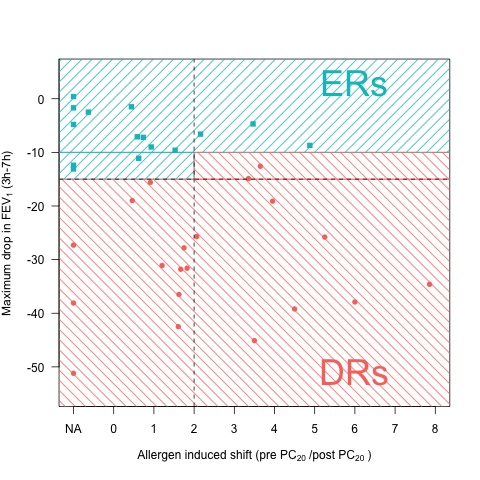


Fig A. Relationship between the maximum drop in FEV_1_ during the late-phase and the allergen-induced shift.

The scatter plot depicts the maximum drop in FEV_1_ during the late-phase (3-7h post-allergen challenge) and the corresponding allergen-induced shift (AIS, ratio between the PC20 from one-day prior to allergen-challenge and the PC20 from one-day after allergen-challenge). A subject is classified as a dual responder if the maximum drop in FEV_1_ during the late-phase is greater than 15%. However, if the maximum drop is between 10-15%, but the AIS is greater than 2, the participant is also classified as a dual responder.


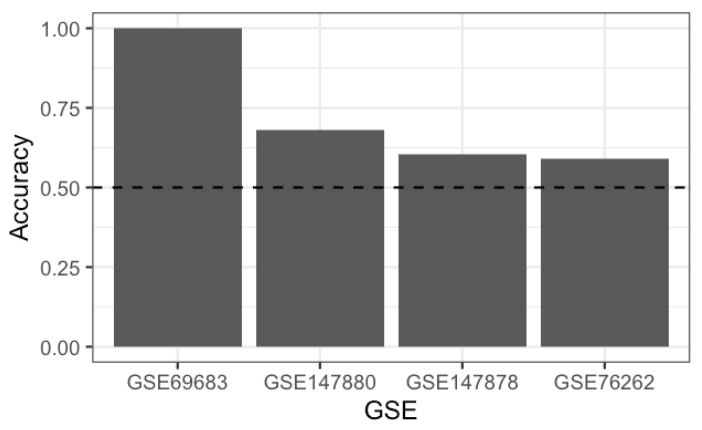


**Fig B. Accuracy of sex imputation in GEO datasets with available biological sex information.** Principal component analysis was performed on Y-chromosome gene expression, followed by k-means clustering (k = 2) on the first two principal components. The cluster exhibiting higher Y-chromosome transcript expression was assigned as male.

**
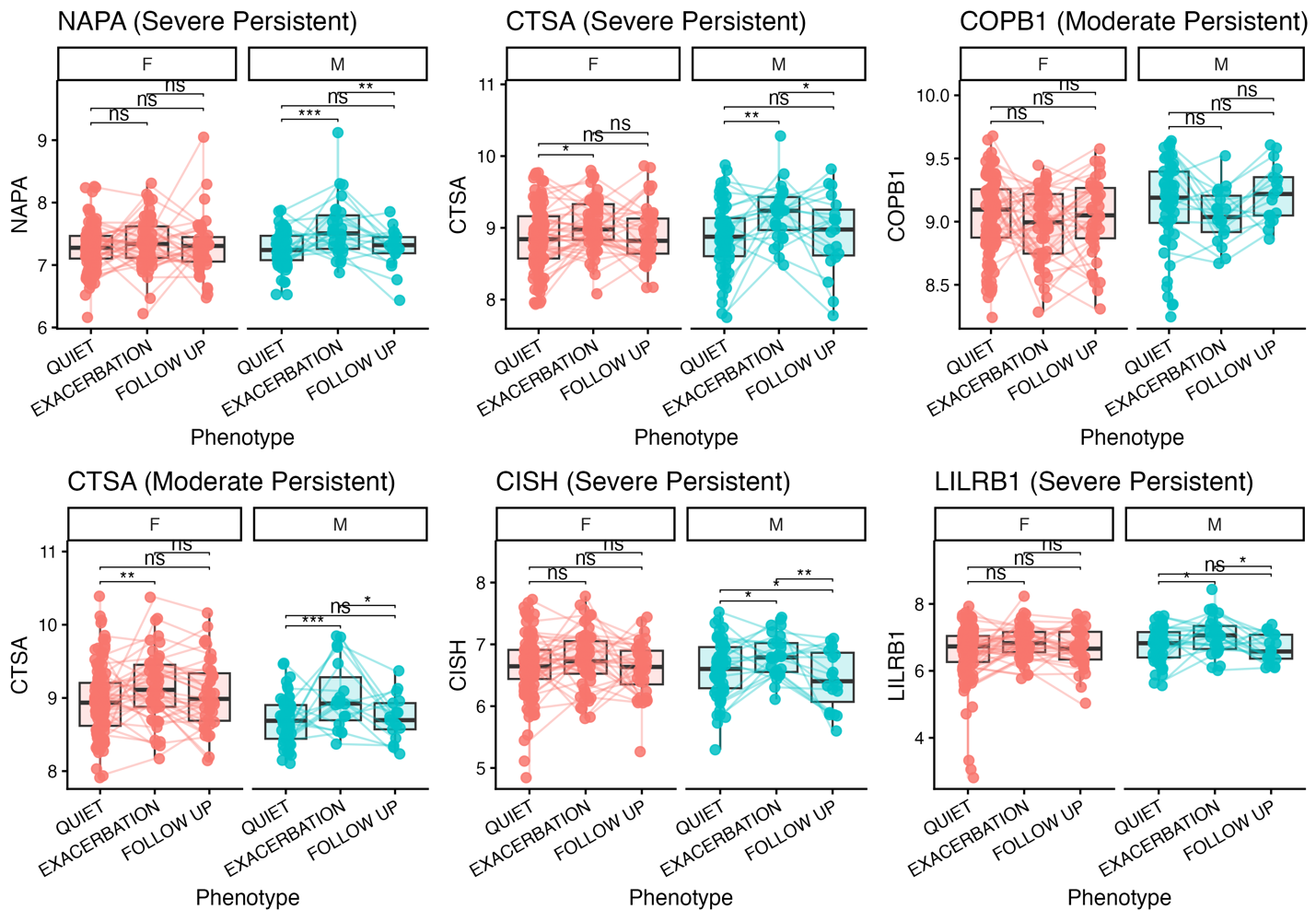
**

**Fig C. Top-ranked biomarkers in male-specific models.** The two highest-ranking transcripts from male-specific models (AUC > 0.70) were examined across multiple comparisons (exacerbation vs quiet and exacerbation vs follow-up) and asthma severities (moderate and severe persistent). Notably, PBMC expression of N-ethylmaleimide-sensitive factor attachment protein (*NAPA*), cathepsin A (*CTSA*), cytokine-inducible SH2-containing protein (*CISH*), and leukocyte immunoglobulin-like receptor B1 (*LILRB1*) increased during exacerbations and decreased during follow-up exclusively in male subjects.


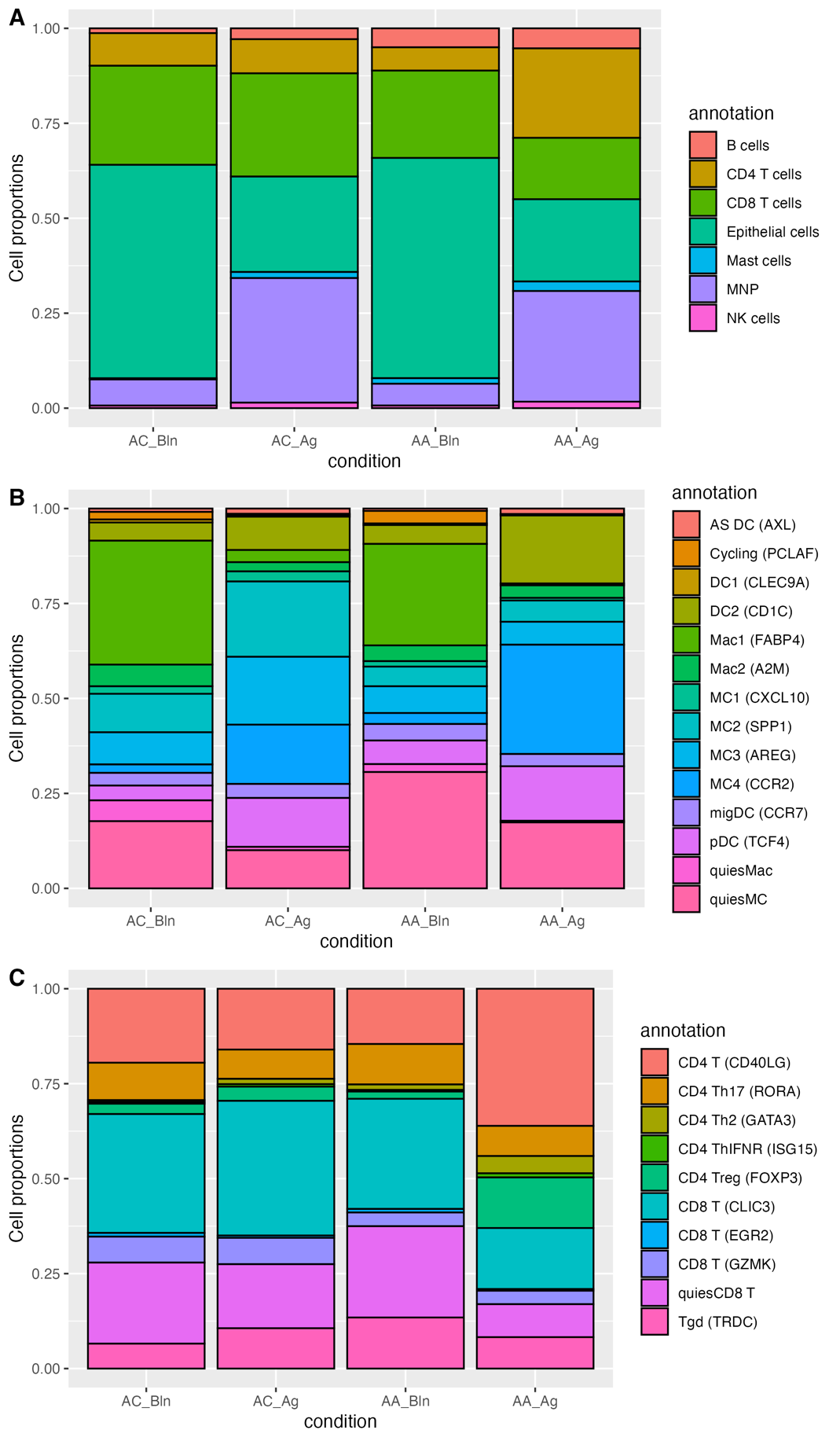


**Fig D. Proportion of cell-types before and after allergen challenge in allergic controls and asthmatics.** AC: Allergic Controls, AA: Allergic Asthma, Bln: baseline, Ag: allergen.

**Supplementary Tables**

**Table A. Number of significant cell-specific genes at a BH-FDR < 20%.**

|  |  | **Allergic controls** | | **Allergic asthma** | |
| --- | --- | --- | --- | --- | --- |
| **Cell-class** | **cell** | **up** | **down** | **up** | **down** |
| **B cells** | **B cells** | 0 | 1 | 26 | 6 |
| **CD4 T cells** | **CD4 T cells** | 2 | 2 | 44 | 10 |
| **CD8 T cells** | **CD8 T cells** | 1 | 2 | 56 | 10 |
| **Epithelial cells** | **Epithelial cells** | 0 | 0 | 26 | 3 |
| **Mast cells** | **Mast cells** | 6 | 2 | 6 | 2 |
| **MNP** | **MNP** | 3 | 3 | 36 | 7 |
| **NK cells** | **NK cells** | 0 | 0 | 17 | 3 |
| **MNP** | **DC2 (CD1C)** | 1 | 0 | 0 | 0 |
|  | **Mac1 (FABP4)** | 1 | 5 | 0 | 0 |
|  | **Mac2 (A2M)** | 0 | 0 | 2 | 2 |
|  | **MC2 (SPP1)** | 6 | 0 | 7 | 5 |
|  | **MC3 (AREG)** | 0 | 0 | 0 | 1 |
|  | **migDC (CCR7)** | 0 | 0 | 0 | 1 |
|  | **quiesMC** | 8 | 2 | 4 | 0 |
| **T cells** | **CD4 T (CD40LG)** | 0 | 0 | 33 | 8 |
|  | **CD4 Th17 (RORA)** | 2 | 2 | 37 | 5 |
|  | **CD4 Th2 (GATA3)** | 0 | 0 | 6 | 1 |
|  | **CD4 Treg (FOXP3)** | 0 | 1 | 0 | 0 |
|  | **CD8 T (CLIC3)** | 11 | 5 | 40 | 4 |
|  | **CD8 T (GZMK)** | 2 | 0 | 13 | 6 |
|  | **quiesCD8 T** | 0 | 0 | 9 | 4 |
|  | **Tgd (TRDC)** | 4 | 1 | 19 | 8 |

**References**

1. Sánchez-Ovando S, Pavlidis S, Kermani NZ, Baines KJ, Barker D, Gibson PG, et al. Pathways linked to unresolved inflammation and airway remodelling characterize the transcriptome in two independent severe asthma cohorts. Respirology. 2022;27:730–8.

2. Shaw DE, Sousa AR, Fowler SJ, Fleming LJ, Roberts G, Corfield J, et al. Clinical and inflammatory characteristics of the European U-BIOPRED adult severe asthma cohort. Eur Respir J. 2015;46:1308–21.

3. Sánchez‐Ovando S, Baines KJ, Barker D, Wark PA, Simpson JL. Six gene and TH2 signature expression in endobronchial biopsies of participants with asthma. Immun Inflamm Dis. 2020;8:40–9.

4. Simpson LJ, Patel S, Bhakta NR, Choy DF, Brightbill HD, Ren X, et al. A microRNA upregulated in asthma airway T cells promotes TH2 cytokine production. Nat Immunol. 2014;15:1162–70.

5. Jia G, Erickson RW, Choy DF, Mosesova S, Wu LC, Solberg OD, et al. Periostin is a systemic biomarker of eosinophilic airway inflammation in asthmatic patients. J Allergy Clin Immunol. 2012;130:647-654.e10.

6. Sun Y, Peng I, Webster JD, Suto E, Lesch J, Wu X, et al. Inhibition of the kinase ITK in a mouse model of asthma reduces cell death and fails to inhibit the inflammatory response. Sci Signal. 2015;8:ra122.

7. Kuo CHS, Pavlidis S, Loza M, Baribaud F, Rowe A, Pandis I, et al. T-helper cell type 2 (Th2) and non-Th2 molecular phenotypes of asthma using sputum transcriptomics in U-BIOPRED. European Respiratory Journal [Internet]. 2017 [cited 2025 Jan 23];49. Available from: https://publications.ersnet.org/content/erj/49/2/1602135

8. GEO Accession viewer [Internet]. [cited 2025 Feb 5]. Available from: https://www.ncbi.nlm.nih.gov/geo/query/acc.cgi?acc=GSE161245
